# Epigenetic Transcriptional Regulation of the *Growth Arrest-Specific gene 1* (*Gas1*) in Hepatic Cell Proliferation at Mononucleosomal Resolution

**DOI:** 10.1371/journal.pone.0023318

**Published:** 2011-08-09

**Authors:** Natalia Sacilotto, Antonio Espert, Josefa Castillo, Luis Franco, Gerardo López-Rodas

**Affiliations:** Chromatin Laboratory, Department of Biochemistry and Molecular Biology, University of Valencia, Burjassot, Valencia, Spain; Bellvitge Biomedical Research Institute (IDIBELL), Spain

## Abstract

**Background:**

*Gas1* (growth arrest-specific 1) gene is known to inhibit cell proliferation in a variety of models, but its possible implication in regulating quiescence in adult tissues has not been examined to date. The knowledge of how *Gas1* is regulated in quiescence may contribute to understand the deregulation occurring in neoplastic diseases.

**Methodology/Principal Findings:**

*Gas1* expression has been studied in quiescent murine liver and during the naturally synchronized cell proliferation after partial hepatectomy. Chromatin immunoprecipitation at nucleosomal resolution (Nuc-ChIP) has been used to carry out the study preserving the *in vivo* conditions. Transcription has been assessed at real time by quantifying the presence of RNA polymerase II in coding regions (RNApol-ChIP). It has been found that *Gas1* is expressed not only in quiescent liver but also at the cell cycle G_1_/S transition. The latter expression peak had not been previously reported. Two nucleosomes, flanking a nucleosome-free region, are positioned close to the transcription start site. Both nucleosomes slide in going from the active to the inactive state and *vice versa*. Nuc-ChIP analysis of the acquisition of histone epigenetic marks show distinctive features in both active states: H3K9ac and H3K4me2 are characteristic of transcription in G_0_ and H4R3me2 in G_1_/S transition. Sequential-ChIP analysis revealed that the “repressing” mark H3K9me2 colocalize with several “activating” marks at nucleosome N-1 when *Gas1* is actively transcribed suggesting a greater plasticity of epigenetic marks than proposed until now. The recruitment of chromatin-remodeling or modifying complexes also displayed distinct characteristics in quiescence and the G_1_/S transition.

**Conclusions/Significance:**

The finding that *Gas1* is transcribed at the G_1_/S transition suggests that the gene may exert a novel function during cell proliferation. Transcription of this gene is modulated by specific “activating” and “repressing” epigenetic marks, and by chromatin remodeling and histone modifying complexes recruitment, at specific nucleosomes in *Gas1* promoter.

## Introduction

The delicate balance between positive signals, that induce cells to enter and progress through the cell cycle, and negative signals, which maintain them in a resting state, controls cell proliferation. Among the models of cell proliferation, the murine partial hepatectomy (PH) offers the advantages of analyzing cell cycle events in a synchronized cell population proliferating in an organism (reviewed in [1], [2]). Although the hepatocytes in adult healthy livers rarely divide, surgical resection, chemical or viral injury have the ability to trigger a regenerative response. The hepatocytes leave the G_0_ state, progress through the cycle and restore the lost hepatic mass. Once this process is completed, cells exit from the cycle to return to reversible growth arrest in an active process that requires growth-inhibitory gene products (reviewed in [2]–[5]).

Special attention has been paid to the identification of genes expressed in quiescent conditions to discover genes that could arrest proliferation of growing cells, for instance, during the development of cancer. Schneider and co-workers [6] analyzed, by subtraction hybridization techniques in quiescent mouse fibroblasts, genes expressed when cells were arrested by serum starvation or contact inhibition. By these means, six cDNA clones were isolated (*Gas1* to *Gas6*). Among these growth arrest-specific (*Gas*) genes, only *Gas1* demonstrated the ability to inhibit cell proliferation when over-expressed in normal and transformed cell lines [7]–[9], and to reduce tumor cell growth [10]–[13]. Apart from these antiproliferative functions, other reported roles for *Gas1* include promotion of apoptosis [10], [14], involvement in mouse embryonic development [15], and suppression of melanoma metastases [16].

Therefore, it seems evident that *Gas1* is a pleiotropic gene, which exerts its functions according to the tissue, the developmental stage or the cellular context. However, no data on the implication of *Gas1* in the maintenance of quiescence of adult tissues, such as liver, are available to date.

The control of eukaryotic cell proliferation requires the integration of several signals that specify a precise transcriptional program, which obviously ought to be developed in a chromatin context (reviewed in [17]–[19]). Epigenetic mechanisms, such as the covalent modifications of DNA and histones, as well as the nucleosomal distribution along the DNA, are strictly regulated, crucial facts that determine the appropriate transcriptional behavior [17], [20]–[25]. Some epigenetic marks have been correlated with transcriptional activation, such as acetylation of histone H3 and H4; or with silencing, such as methylation of cytosines at the CpG islands and of some histone residues (reviewed in [22], [24], [25], [26]). However, it is more likely that a specific combination of marks, acting in a complex network of interactions, drive the transcriptional response in particular cell types or environments. Therefore, understanding how proliferation of normal cells is epigenetically controlled is a pre-requisite to define the mechanisms of deregulation of cellular behavior, such as in cancer development.

To better know how the transcriptional regulation of *Gas1* is carried out under controlled proliferation, we analyze in this work the expression of the gene during mouse liver regeneration, a process in which cells proliferate synchronously. We find that *Gas1*, expressed in quiescent liver, is repressed when the cells enter the cell cycle, to be again expressed at the G_1_/S transition. The epigenetic marks and the changes in nucleosomal positioning around the transcriptional start site have been studied and some distinctive features of the repressed state and of both the active ones are described. Our results suggest that the mechanisms regulating *Gas1* transcription are different at a chromatin level in both active states.

## Results

### The *Gas1* gene shows a biphasic pattern of gene expression in adult liver after PH

**Figure 1:**
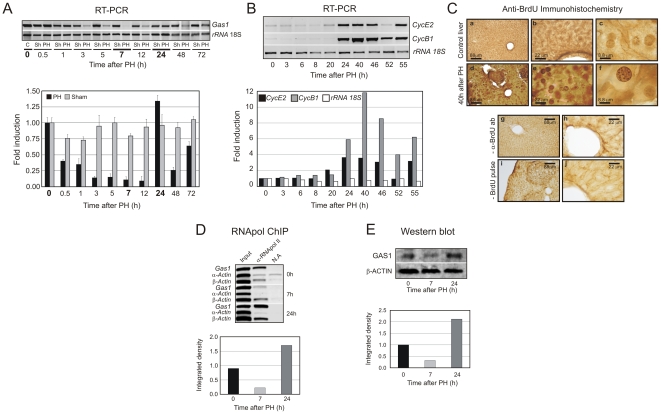
Expression of *growth arrest gene-1* (*Gas1*) during mouse liver regeneration after partial hepatectomy (PH). (A) Steady state mRNA levels of *Gas1* were measured by semiquantitative (top) and quantitative (bottom) RT-PCR in liver at the indicated times after PH. The values were normalized by the loading control *rRNA* 18S and expressed as relative to control liver at 0 h after PH. Mice that underwent only laparotomy (Sham operation) were used as control. The bars of errors correspond to the standard deviation of 5–6 independent RT-PCR measurements. (B) Expression of cyclines *CycE2* and *CycB1*. Steady state mRNA levels of cyclines were measured by semiquantitative RT-PCR (top panel) and the bands were integrated by ImageJ software analysis (bottom panel). The values were normalized by the loading control *rRNA* 18S and expressed as relative to control liver. (C) Immunohistochemical detection of BrdU incorporation at 0 h and 40 h after PH. Immunostaining negative control, with tissue treated in absence of primary α-BrdU antibody, and in absence of BrdU pulse, is also shown. These images are representative of three different experiments. (D, E) Expression of *Gas1* at the two selected transcriptional active states (0 and 24 h) and at repressed state (7 h) after PH. The measure was done by RNApol ChIP assay (D) and Western blotting (E). The bands were integrated by ImageJ software and the histograms (bottom panels) were normalized against control liver. The *α-* and *β-Actin* genes were used as negative and positive control respectively, and *rRNA* 18S as an internal control of the RT-PCR analysis.

Although extensive information implicating *Gas1* in growth control of different cell types is available, no data on the possible involvement of *Gas1* in regulating cell proliferation in adult tissues have been reported to date. The adult liver is mainly a quiescent organ and *Gas1* has been described as a quiescence gene marker *in vitro* [6]. We first analyzed *Gas1* expression pattern during liver regeneration after partial hepatectomy (PH) in mice. The semi-quantitative and quantitative RT-PCR (Figure 1A), show that *Gas1* is expressed in control livers (0 h, non-operated mice) and that its expression decreases rapidly to reach a minimum at around 7 h after PH. The gene also shows an unexpected maximum of expression at 24 h after PH, a time at which hepatocytes are about to enter S-phase, as estimated by analysis of the induction of cell cycle markers *CycE2* and *CycB1* (Figure 1B), and by BrdU incorporation (Figure 1C).

We also analyzed *Gas1* expression pattern in sham-operated mice, since surgical stress, even without liver resection, could induce physiological responses that either activate or silence the expression of many genes. The results show that *Gas1* expression is not altered by surgical stress itself (Figure 1A).

These data suggest that GAS1 may have a new potential function during the G_1_/S transition since, according with its classical function in quiescence, it was not expected an increase of gene expression in liver regeneration until the end of the first round of division when cells start to arrest in a reversible G_0_ state.

Since not only did we find *Gas1* expression during quiescence, but also unpredicted up-regulation of the gene during the G_1_/S transition in liver, we focused our interest in studying the regulation of *Gas1* in these transcriptional active states in a chromatin context. We further confirmed that *Gas1* was actively transcribed by RNApol ChIP (Figure 1D) and that the protein was synthesized (Figure 1E), at 0 and 24 h after PH. On the contrary, the RNApol II was not present at the coding region of the gene (Figure 1D), and protein level was reduced significantly, at 7 h after PH (Figure 1E).

### Analysis of nucleosome positioning at the *Gas1* promoter

As we have pointed out before, eukaryotic gene transcription is regulated in a chromatin context, so we wondered whether the changes in *Gas1* transcription during liver regeneration are accompanied by changes in chromatin structure. Taking into account that remodeling of chromatin at the promoters is a feature of most inducible genes (see recent reviews in [27]–[29]), we first analyzed, by classical ChIP assay, the recruitment of the main chromatin remodeling complexes using antibodies against components of the SWI/SNF (BRM or BRG1), ISWI (SNF2 h) and CHD (MTA1) families of remodelers (Figure S1). The results indicated that none of the analyzed remodeling complexes is bound to *Gas1* promoter in the inactive state (7 h after PH), while in the active state during liver quiescence, the SNF2 h-containing ISWI remodeling complex appears bound to the promoter. In the second active state, at 24 h after PH, a BRM-containing remodeler binds the promoter. These results strongly suggest that a remodeling of the promoter chromatin is required for *Gas1* to be expressed, and this process is accomplished in different ways in both transcriptionally active states. The other complexes checked, i.e., those containing BRG1 or MTA1, do not participate in these remodeling events under these conditions.

**Figure 2:**
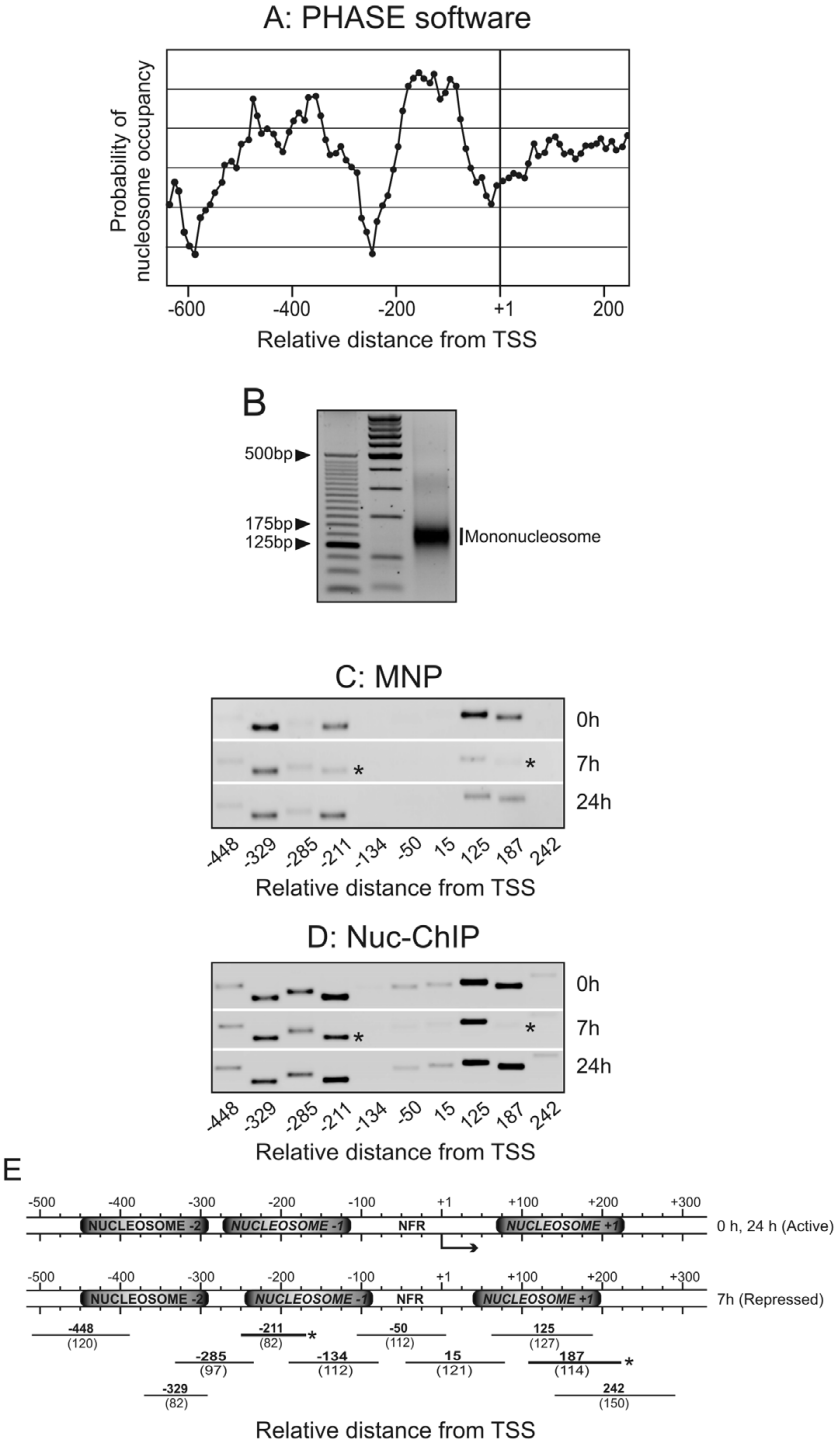
Nucleosome positioning at *Gas1* transcriptional start site (TSS) vicinity. (A) Theoretical prediction of nucleosome positioning based on PHASE software algorithms. (B) Agarose gel showing mononucleosomal-sized chromatin fragments used for nucleosome positioning. PCR analysis from (C) micrococcal nuclease protection (MNP) and (D) Nuc-ChIP assays for nucleosome positioning. (E) Schematic representation of the positions of nucleosomes empirically determined. The positions of the center (bold letters) and lengths (brackets) of the amplicons used in the PCR reactions are showed at the bottom of the panel. The images are representative of at least three independent experiments.

We next determined, in the inactive state and in both the active ones, the position of nucleosomes near the transcriptional start site (TSS). First, the theoretical prediction of nucleosome positioning near the TSS, based on the PHASE program algorithm (available in http://wwwmgs.bionet.nsc.ru/mgs/programs/phase/), showed a high probability of nucleosomal presence between −50 to −200 from TSS, a moderate probability between −300 to −500, and a low probability between +50 to +200 (Figure 2A). In view of this prediction, we designed a series of primers giving tiled amplicons between −500 and +300 to examine the nucleosome positioning. To do this, two methodologies were used, namely the micrococcal nuclease protection (MNP) assay and the mononucleosomal immunoprecipitation (Nuc-ChIP) assay. The first method consisted in an extensive digestion of crosslinked liver nuclei with micrococcal nuclease, followed by DNA purification and isolation of the mononucleosomal DNA band from an agarose gel (Figure 2B). The second method consisted in an immunoprecipitation of mononucleosomal-sized chromatin fragments using an antibody against the histone H3, with the consequent DNA isolation. In both cases, the DNA was further used as a template in PCR reactions using the designed primers.

The results obtained from both MNP assay (Figure 2C) and Nuc-ChIP assay (Figure 2D) showed a strong signal for the amplicons located at −329, at −211 and at +125 /+187, that should correspond to three positioned nucleosomes, further referred to as N-2, N-1 and N+1 (Figure 2E). The data also suggest that N+1 and N-1 nucleosomes may experience a sliding during the transition between the actively transcribed and the repressed state, since the signals of the amplicons −211 and +187 are significantly lower at 7 h in comparison with 0 and 24 h after PH (labeled with asterisks in Figure 2C, 2D and 2E). On the contrary, the nucleosome N-2 seems to be located at the same position, within the resolution limits of this experiment, in the three transcriptional states of *Gas1*, since the signal at the amplicon −329 bp is similar at 0 h, 7 h and 24 h after PH.

**Figure 3:**
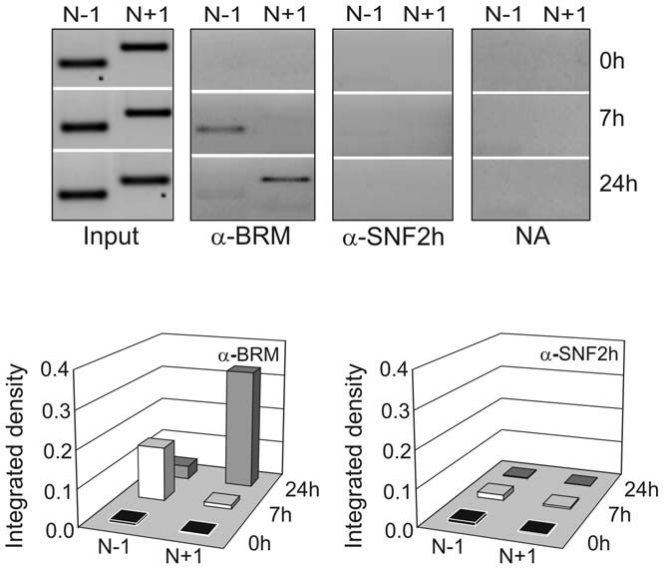
Nuc-ChIP assay of BRM-containing SWI/SNF and SNH2 h-containing ISWI remodeling complexes after PH. The PCR bands were integrated by ImageJ software, the background (no antibody samples, NA) subtracted, normalized by dividing by their corresponding Input samples and represented as histograms at the bottom panel. The images are representative of at least three independent experiments.

The above results encouraged us to determine which chromatin remodeling complexes could be implicated in the sliding of nucleosomes N-1 and N+1 during the transition between the different transcriptional states of *Gas1*. To address this issue, a Nuc-ChIP experiment was carried out. Our results (Figure 3) showed that BRM-SWI/SNF complex was bound to N-1 when *Gas1* is repressed at 7 h after PH, and very weakly at 24 h post-HP. Moreover, this complex was strongly bound to nucleosome N+1 in G_1_/S transition at 24 h post-HP. On the contrary, SNF2 h-ISWI complex was bound neither to N-1 nor to N+1 at the three analyzed *Gas1* transcriptional states. The functional importance of the nucleosomal remodeling of the *Gas1* TSS vicinity by BRM-SWI/SNF is not yet clear, but it might be related to the fact that some transcriptional factors, namely CREB, C/EBPβ, SMAD4 or SP1, are bound to their transcriptional binding sites located near these nucleosomes, as it was assessed by standard ChIP assay using antibodies against these factors (Figure S2).

### HAT and HDAC recruitment to *Gas1* promoter at mononucleosomal resolution

To explore the epigenetic regulation of *Gas1,* we carried out analyses of the recruitment of histone acetyltransferases (HAT) and histone deacetylases (HDAC) to the gene promoter by chromatin immunoprecipitation (ChIP) assay at mononucleosomal resolution. We again selected the two transcriptional active states of *Gas1*, quiescence and G_1_/S transition, and the transcriptional inactive state, at 7 h after PH.

The preliminary studies, using standard ChIP assay, indicated that GCN5 was bound at the *Gas1* promoter in quiescent liver. GCN5 binding was less clear at 7 h after PH and it was released from the promoter at the G_1_/S transition. By contrast, CBP is constitutively bound to *Gas1* promoter at the three selected time-points. The other HAT complexes analyzed, p300 and PCAF, do not seem to be implicated in the acetylation pattern of *Gas1* promoter in the time-points under study (data not shown). In relation with the HDACs, mSIN3A was bound to *Gas1* promoter in quiescence and at 7 h, and the ChIP analysis only gave a faint band 24 h after PH. It is also noteworthy that the NCoR-containing histone deacetylase complex was constitutively bound to *Gas1* promoter at the three selected transcriptional states (data not shown).

**Figure 4:**
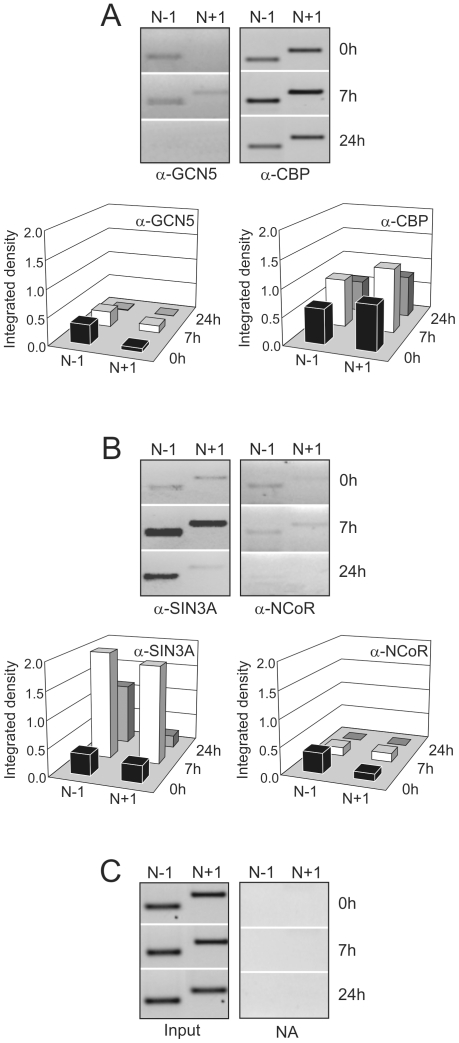
Nuc-ChIP assay of the chromatin modifying complexes recruitment to *Gas1* TSS vicinity after PH. (A) histone acetyltransferase (GCN5-containing and CBP-containing) and (B) histone deacetylase (Sin3A-containing and NCoR-containing) complexes. (C) Input and non-antibody (NA) samples used to subtract the background and to normalize PCR band signals as described in figure 3.The images are representative of at least three independent experiments.

Consequently, we analyzed the recruitment/release of those histone modifying complexes to the specific nucleosomes located at the *Gas1* TSS vicinity. Figure 4A shows that GCN5 is bound to nucleosome N-1 in quiescent liver and at 7 h after PH. As to nucleosome N+1, it only binds GCN5 in the inactive state (7 h after PH). On the contrary, CBP is bound to both nucleosomes at the three transcriptional states studied.

On the other hand, the mSIN3A-containing HDAC complex was actively recruited at 7 h post-HP, when *Gas1* is repressed. Afterwards, the SIN3A complex remains bound to nucleosome N-1, although it was released from nucleosome N+1 in the G_1_/S transition, when *Gas1* is again active (Figure 4B). The binding of NCoR-containing HDAC complex to *Gas1* promoter displays a pattern roughly similar to that of GCN5 (Figure 4B).

### Epigenetic marks in *Gas1* promoter at mononucleosomal resolution

**Figure 5:**
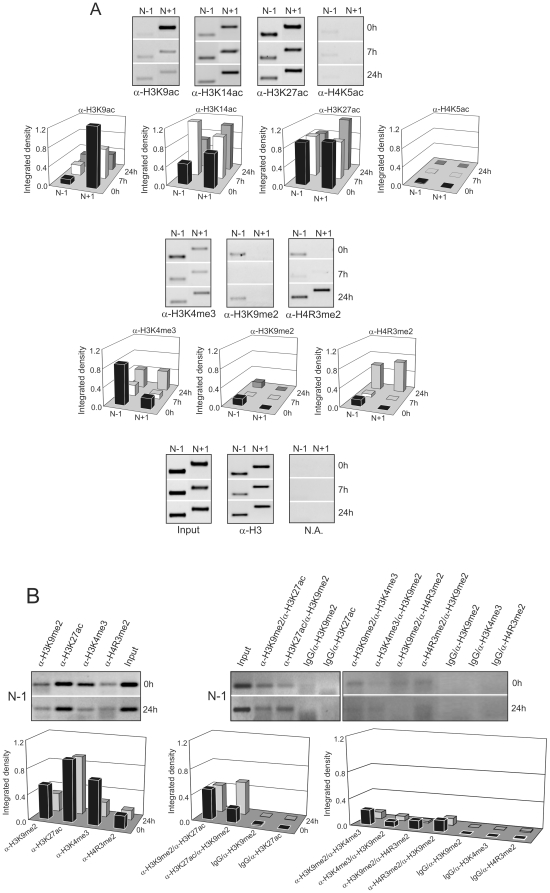
Nuc-ChIP and sequential-ChIP assays of site-specific epigenetic marks on nucleosomes near to *Gas1* TSS vicinity. (A) Histone acetylation and histone methylation marks at nucleosomes N-1 and N+1 of *Gas1* promoter at 0 h, 7 h and 24 h after PH. The PCR bands were integrated by ImageJ software and corrected as in figure 3. The images are representative of at least three independent experiments. (B) Sequential ChIP analysis of “inactivating” (H3K9me2) and “activating” (H3K27ac, H3K4me3 and H4R3me2) epigenetic marks at nucleosome N-1 of *Gas1* promoter. Both orders for first and second antibody are assayed. The PCR bands were integrated by ImageJ software and corrected as in figure 3. To subtract the background, the integrated density of a sample where first antibody was replaced by IgG was used. The images are representative of at least three independent experiments.

We next analyzed if there are distinct epigenetic marks in the positioned nucleosomes located at the *Gas1* TSS vicinity in the three transcriptional states. To perform this analysis, the crosslinked fragments of chromatin of mononucleosomal size, obtained from micrococcal nuclease digestion, were immunoprecipitated with antibodies against specific histone modifications (Figure 5A).

We found different patterns of histone modifications in the nucleosomes under the diverse conditions studied. For instance, H3K9ac seemed to be a specific mark of N+1 at 0 h; H4R3me2 appeared at 24 h in the N-1 and N+1 nucleosomes; H3K4me3 appeared on N-1 at 0 h and 24 h. The H3K14ac and H3K27ac were present with higher or lower intensity at both nucleosomes in the three analyzed time-points.

These results suggest that the regulation of the epigenetic marks level along the *Gas1* TSS vicinity is rather complex, since there is combination of “inactive characteristic marks” (as H3K9me2) and “active characteristic marks” (as H3K9ac, H3K14ac, H3K27ac, H4K5ac, H3K4me3 or H4R3me2) not only in neighbor nucleosomes, but also within the same nucleosome. Some “activating” marks are present at both analyzed nucleosomes when *Gas1* is transcriptionally active in quiescence and in G_1_/S transition. It is noteworthy that some distinctive, specific marks have been found at the different transcriptional states, as it happens with the presence of H3K9ac in nucleosome N+1 in quiescence or with H4R3me2 in nucleosomes N-1 and N+1 in the G_1_/S transition.

The presence of “inactivating” and “activating” epigenetic marks within the same nucleosome could be due to a monoallelic gene expression of *Gas1* as an additional mechanism to regulate precisely the level of transcription in response to cellular stimuli during quiescence and G_1_/S transition. By this mechanism, the hepatocyte could maintain the desired level of transcript depending on whether one or two alleles are transcribed and, at the same time, the transcriptional activity of each allele may be epigenetically regulated through independent ways. Gimelbrant and co-workers [51] performed a genome-wide search for genes subject to monoallelic expression and found that up to 1000 genes in human genome may be subject to such monoallelic expression, including not only X-inactivated genes in development or autosomal imprinted genes but also other autosomal genes. Among the latter class, these authors found *Gas2* and *Gas6*, members of the growth arrest-specific gene family, as genes that are expressed monoallelically.

To check if the “activating” and “repressing” marks are coexisting in *Gas1* promoter in the same nucleosome, but being located at different alleles, we perform a sequential-ChIP analysis (also known as re-ChIP) at nucleosome N-1 by combining Nuc-ChIP assay, using an antibody against H3K9me2, followed by reimmunoprecipitation with an antibody against H3K27ac, H3K4me3 or H4R3me2, and *vice versa*.

The results represented in Figure 5B indicate that indeed both types of epigenetic marks are located not only at the same nucleosome but also in the same allele since different combination of “activating” and “repressing” epigenetic marks in the sequential-ChIP showed significant band signals as evaluated by the ImageJ software (Figure 5B, bottom panel).

## Discussion

In spite of the extensive information on the role of the *Gas1* in cell growth control *in vitro*, in programmed cell death and in mouse development *in vivo*, no data on the role of *Gas1* in the quiescence of adult tissues is available. On the other hand, over-expression of *Gas1* inhibits cell proliferation in different tumor cell lines [10], [31], [32], as well as in cultured fibroblasts [9] and, recently, the possible benefits of *Gas1* in tumor gene therapy have been advanced [12], [16].

In the present study, using the natural synchrony of the cell proliferation model after partial hepatectomy, we describe that *Gas1* is expressed not only in quiescent liver but also actively transcribed just before entering S phase. This is the first time that expression of *Gas1* at that moment of the cell cycle is described, because, to date it has only been showed expression of the gene when cell lines are arrested in G_0_ [1], [2], [4], [5], [19]. Our data suggest that *Gas1* expression could be playing a dual role in liver cell cycle control, acting to keep liver quiescence, and to control the pass of the cells through the G_1_/S checkpoint. The expression of *Gas1* would facilitate the arrest of the cell cycle before entering S phase if cell damage has occurred. In this context it should be noted that overexpression of *Gas1* in cultured cells causes arrest of the cell cycle at the interface G_1_/S [7], [9], [33]. Further research is obviously needed to ascertain the molecular mechanisms involved.

We have described that *c-Myc*, which is expressed during liver regeneration in a biphasic manner, is distinctly activated in both expression waves [34], so we wondered whether the mechanisms involved in the transcriptional regulation of *Gas1* in quiescence and in the G_1_/S transition are also different.

It has been described that c-MYC and v-Src-repressed expression of *Gas1* is not linked to the TGF-β signalling pathway [35], [36]. c-MYC also regulates several cell cycle regulatory genes as *p15*, *p21*, *p27*, *Gadd34*, *Gadd45*, and *Gadd153*. c-MYC represses transcription of these genes by at least two distinct mechanisms. One mechanism requires DNA binding of the MYC–MAX complex to Inr element in their promoters, and the inhibition of transcriptional activators via the C-terminal domain of c-MYC. The other mechanism is dependent on c-MYC binding to the SP1 transcription factor via the c-MYC central region inhibiting SP1 transcriptional activity (see review in [37]). We have found that SP1 is present at *Gas1* promoter under repression at 7 h after PH (Figure S2), suggesting that this second mechanism may be acting in *Gas1* repression during liver regeneration after PH. Comparative studies carried out by de Martin and co-workers by EMSA technique using growing and resting NIH3T3 cell extracts suggested that most of the growing and repressing state-specific protein-binding sites are located between nucleotides −195 and −550 [38]. This region is highly conserved from chicken to human, as assessed by *Gas1* promoter alignment (http://genome.lbl.gov/vista/index.shtml) (data not shown), and contains several putative binding sequences for SP1 binding (as estimated by TESS software http://www.cbil.upenn. edu/cgi-bin/tess/tess) (data not shown). Nevertheless, it cannot be ruled out that other mechanisms might be also acting in *Gas1* transcriptional regulation since it has been reported that MYC-box 2 (MB2) and leucine repeat C-terminal region (LZ) of c-MYC are required for *Gas1* repression in Myc-transformed Rat-1a fibroblast cell lines [35], regions that do not interact with SP1 transcriptional factor [37].

In view of the importance of chromatin structure in eukaryotic gene regulation, we focused our interest on the changes in chromatin when going from one transcriptional state to another. First, we have determined that three nucleosomes, N-2, N-1 and N+1, are positioned in the neighborhood of the TSS. A nucleosome-free region (NFR) is flanked by nucleosomes N-1 and N+1. These results are in agreement with the findings obtained by genome-wide mapping which have revealed that N-1 and N+1 nucleosomes delimiting a NFR is an evolutionarily conserved, common feature of promoters. In our case, both nucleosomes reside in canonical locations at specific distances from the TSS [39]–[44]. As “gate-keepers” of the NFR at the promoters, the N-1 and N+1 nucleosomes are well positioned to have significant regulatory potential in transcription. Published data show that, when genes are transcribed, nucleosome N+1 is shifted downstream of TSS [40], [43], as it happens to *Gas1* nucleosome N+1 in quiescence and G_1_/S transition (Figure 2). The observed sliding probably leaves the TSS fully accessible for the transcriptional machinery (reviewed in [43]). In our case, the data are also compatible with an upstream sliding of nucleosome N-1 upon transcription of the gene (Figure 2). This sliding may be related to changes in the accessibility of transcriptional factors, as CREB, C/EBPβ, SMAD4 or SP1, to their sequence-specific binding sites located near N-1 nucleosome, as we estimated by TESS software and analyzed by ChIP assay (Figure S2).

It is noteworthy that a differential binding of the BRM catalytic subunit to N-1 and N+1 nucleosomes occurred at the analyzed transcriptional states. The data of Figure 3 indicate that the remodeling complex is mainly bound to nucleosome N-1 at 7 h and to nucleosome N+1 at 24 h after PH. That complex could be responsible of the N-1 sliding when going from the active state in quiescent liver to the repressed state at 7 h after PH and for the transition to the second active state at 24 h after PH. On the contrary, the complex does not seem to participate in the sliding of nucleosome N+1 when going from the inactive state to the active one at 24 h after PH (Figure 2). Although the ISWI complex is bound to the active promoter, as revealed by the binding of its component SNF2 h detected by low-resolution standard ChIP assay, we have not found evidence for the association of ISWI complex to N-1 or N+1 nucleosomes. It is possible that this complex acts on some neighbor nucleosomes not studied in the present work. Of course, other non-studied remodeling complexes may participate in the remodeling events of *Gas1* promoter and proximal transcribed region.

There is a plethora of data showing that covalent histone modifications play a crucial role in transcriptional regulation (see reviews in [17], [23], [26], [45]–[51]). However, information about histone modifications at specific nucleosomes in different transcriptional states is limited in the literature and this circumstance adds some interest to the data presented in Figures 4 and 5. As Figure 4 shows, GCN5-containing HAT complex is bound to nucleosome N-1 in quiescence, and to N-1 and N+1 nucleosomes at 7 h post-HP. On the contrary, CBP is constitutively bound to these nucleosomes flanking *Gas1* TSS at the three transcriptional states. On the other hand, the mSIN3A-containing HDAC complex is mainly bound to the analyzed nucleosomes when *Gas1* is repressed at 7 h post-HP, while the low signal obtained for the binding of NCoR-containing HDAC complexes make it difficult the drawing of definite conclusions. At any rate, the present data suggest that the regulated acquisition of histone epigenetic marks depends on the balanced and controlled activities of the opposite histone modifying enzymes activities acting over the same specific nucleosome.

The pattern of histone modifications in N-1 and N+1 nucleosomes is rather complex (Figure 5). We were able to distinguish different “categories” of modifications. For instance, modifications such as H3K9ac and H3K27ac are constitutively present in both analyzed nucleosomes at the three transcriptional conditions and they might be related to the generation of a relaxed chromatin structure at *Gas1* promoter as expected for a gene poised for transcription. On the other hand, the modifications H3K9ac and H3K4me2 are mainly related with quiescence, whereas H4R3me2 is mainly related with G_1_/S transition. Those modifications could be participating in the acquisition of a platform to which regulatory proteins may bind chromatin to activate or repress transcription of *Gas1* in response to a specific cellular signaling.

We found that nucleosome N-1 at *Gas1* promoter at 0 h and 24 h after PH contains not only “activating” (H3K27ac, H3K4me3 and H4R3me2) epigenetic marks, as expected since the gene is transcriptional active, but also the known “inactivating” mark H3K4me2. The widespread monoallelic expression analysis on human autosomes, carried out by Gimelbrant and co-workers [30], showed that both *Gas2* and *Gas6*, two members of the growth arrest-specific gene family, belong to the group of genes expressed monoallelically. This finding opens the hypothesis that *Gas1* could be differentially regulated in both alleles by differential epigenetic marks leading to a monoallelic gene expression. However, the results obtained by sequential-ChIP (Figure 5) demonstrate that this does not occur in *Gas1* since both types of epigenetic marks colocalize within the same nucleosome at *Gas1* promoter.

There is a growing body of data in the literature showing that “repressing” H3K9 methylation is distributed at both silent heterochromatin and at active genes [52]–[56]. Wiencke and co-workers [54], using ChIP-on-chip experiments and conventional ChIP assay, confirmed the positive associations of H3K9me3, H3K4me2 and H3K9ac modifications with gene expression. The data also revealed that H3K9 methylation overlaps with those histone “activating” marks at TSS surrounding of genes. The information obtained in our studies indicate that the “inactivating” H3K9 methylation may colocalize, at least at nucleosome N-1, with several activating marks. It can not be ruled out the possibility that H3K9me2 is located in one of the H3 molecule and the “activating” marks on the other H3 molecule of the nucleosome, but the lack of specific antibodies for H3 against dual H3K9me2 and “activating” H3 marks leave open the question as to whether both modifications occur on the same histone H3 tail.

The global analysis of the data presented in this paper, together with those showed by other authors [52]–[56], suggests the need of revising aspects of the histone code involving plasticity of H3 lysine methylation and its “exclusive” association with repressed genes.

## Materials and Methods

### Biological material and experimental design

All procedures were conducted in accordance with the European regulations (Council Directive 86/609/EEC) and were authorized by the Ethics Committee for Animal Experimentation of the University of Valencia (Approval of procedure for project BFU2007-63120, date 12/03/2007). Eight to ten week-old CD1 male mice were used in the experiments. The partial hepatectomy (PH) technique was based on the classical method described by Higgins and Anderson [57]. Briefly, mice were anaesthetized with isofluorane and the median and left lateral liver lobes (2/3 of liver mass) were excised. Mice were sacrificed under anesthesia at the indicated time-points after PH, and the remnant livers were harvested for RNA, protein and chromatin extraction. Mice that underwent only laparotomy (sham operation) were used as control.

### RNA extraction, RT-PCR and qRT-PCR

The livers were harvested from anaesthetized mice and snap-frozen in liquid nitrogen. After disrupting 30 mg of tissue in an Ultra-Turrax homogenizer (IKA Werke, Staufen, Germany), RNA was extracted and purified with the Illustra RNAspin Mini RNA Isolation Kit (GE Healthcare, Pittsburgh, PA, USA) according to the manufacturer's instructions. The RNA concentration was estimated by measuring the absorbance at 260 nm, and its purity double-checked by calculating the ratio A_260_/A_280,_ and by visualizing RNA on agarose formaldehyde gel electrophoresis. 1 µg of total RNA was retrotranscribed to cDNA using Superscript II RNase H^-^ (Invitrogen, Carlsbad, CA, USA), following the manufacturer's instructions, with random hexamers (Sigma, St. Louis, MO, USA) to prime the elongation reaction. cDNA was analyzed by PCR, the products were size-fractionated by 2% agarose gel electrophoresis, stained with ethidium bromide, photographed with Gel Doc XR^+^ image analyzer (Bio-Rad, Hercules, CA, USA) and quantified by ImageJ software (http://rsbweb.nih.gov/ij/). The quantitative real time RT-PCR (qRT-PCR) were conducted in an ABI GeneAmp 7000 Sequence Detection System (Applied Biosystems, Carlsbad, CA, USA) and analyzed with the ABI Prism Software (Applied Biosystems), and the relative expression values were calculated according to the 2^–ΔΔCt^ method.

The primer pairs used for RT-PCR were: *Gas1,* forward 5′-CGAACACTGCAGGTCCACCAAG-3′, reverse 5′-TCGCACACGCAGTCGTTGAG-3′, amplicon position +937/+1209 and size 273 bp; *18S rRNA,* forward 5′-TGGTTGATCCTGCCAGTAGC-3′, reverse 5′- CTCTCCGGAATCGAACCCTG-3′, amplicon position +1327/+1580 and size 254 bp. The primer pairs used for qRT-PCR: *Gas1,* forward 5′-TGGATGAGGACGCCCATG-3′, reverse 5′- GGAACTCGGA CAAACTTTTCCA-3′, amplicon position +426/+477 and size 52 bp; *18S rRNA,* forward 5′-CACGGCCGGTACAGTGAAA-3′, reverse 5′-AGAGGAGCGAGCGACCAA-3′, amplicon position +67/+138 and size 72 bp.

### Protein extraction and western blot

Fragments of 100 mg of liver were homogenized in 1 ml of ice-cold PBS, supplemented with 2 µl of protease inhibitor cocktail (Sigma). After centrifuging and removing PBS, total protein extraction was performed in RIPA buffer (50 mM Tris-HCl, 150 mM NaCl, 1% Nonidet P-40, 0.5% sodium deoxycholate, 0.1% SDS, 2 µl/ml protease inhibitor cocktail, pH 8), keeping the samples 2 h under rotation at 4°C. The lysates were centrifuged at 14,000*xg* for 10 min at 4°C, and supernatants containing the soluble proteins were recovered. Protein quantification was performed with the Protein Assay Reagent kit (Bio-Rad) according to the manufacturer's instructions.

Thirty µg of total protein extracts were run in 12% SDS-PAGE and transferred to a 0.45 µm-pore nitrocellulose membrane, incubated with primary antibodies [goat α-GAS1 (R&D Systems, AF2644), or goat (-β-ACTIN (Abcam, ab8229)], washed and incubated with rabbit anti-goat HRP-conjugated secondary antibody (Bio-Rad, 172-1034) and developed with ECL advance detection kit (GE Healthcare), according to the manufacturer's instructions.

### ChIP, Nuc-ChIP, RNApol ChIP and sequential ChIP

The ChIP and RNApol ChIP procedures were performed according to Sandoval and co-workers [58], [59], and Rodriguez and co-workers [33], [60] with some modifications. Livers were surgically removed from anaesthetized animals, washed in PBS and fixed by immersion in 1% formaldehyde in PBS under rotation during 15 min at room temperature. The cross-linking reaction was stopped by adding glycine to a final concentration of 0.125 M, and incubating for 5 min under rotation at room temperature. Afterwards, samples were washed twice in ice-cold PBS and homogenized in a Potter-Elvehjem tissue grinder with 10 ml of ice cold PBS supplemented with 2 µl/ml protease inhibitors cocktail (Sigma). The tissue homogenate was filtered through a 500 µm pore nylon membrane and centrifuged at 3,000xg for 5 min. The cell pellet was resuspended in 6 volumes of cell lysis buffer (10 mM NaCl, 3 mM MgCl2, 30 mM sucrose, 10 mM EDTA, 0.5% Nonidet P-40, 10 mM Tris-HCl, pH 7), supplemented with 2 µl/ml protease inhibitor cocktail (Sigma), and incubated on ice during 15 min. After incubation, the suspension was centrifuged at 1,000xg for 5 min, and the nuclear pellet was resuspended in 6 volumes of cell lysis buffer. After centrifuging at 1,000xg for 5 min, the supernatant was carefully removed, and the nuclear pellet were resuspended in 1 volume of nuclei lysis buffer (10 mM EDTA, 1% SDS, 50 mM Tris-HCl, pH 8.1) and stored at −80°C until use.

Cross-linked chromatin was disrupted on ice with 8 pulses, 10 s each, in a Vibra-Cell VCX-500 sonicator (Sonics and Materials, Newtown, CT, USA) to obtain chromatin fragments of 300–500 bp. The fragmented chromatin was centrifuged at 14,000*xg* for 10 min, and the supernatants were 10-fold diluted with dilution buffer (165 mM NaCl, 0.01% SDS, 1.1% Triton X-100, 1.2 mM EDTA, 16.7 mM Tris–HCl, pH 8.0) supplemented with protease inhibitor cocktail (Sigma). Aliquots from the diluted supernatants (equivalent to 50 µg DNA) were incubated under rotation for 2 h at 4°C with Dynabeads-protein G (Invitrogen) and 2 µg of the corresponding antibodies. The chromatin fragment/antibody/protein G-Dynabead immunocomplexes, were recovered and washed twice with ice-cold low-salt buffer (140 mM NaCl, 1% Triton X-100, 0.1% sodium deoxycholate, 1 mM EDTA, 50 mM Tris-HCl, pH 8.0), twice with ice-cold high-salt buffer (500 mM NaCl, 1% Triton X-100, 0.1% sodium deoxycholate, 1 mM EDTA, Tris-HCl 50 mM, pH 8.0), twice with ice-cold LiCl buffer (250 mM LiCl, 0.5% NP-40, 0.5% sodium deoxycholate, 1 mM EDTA, Tris–HCl 10 mM, pH 8.0) and finally once with TE buffer. The immunoselected chromatin was eluted from the Dynabead-protein G in two consecutive steps, by adding 50 µl of elution buffer (EDTA 10 mM, SDS 1%, 50 mM Tris–HCl), vortexing and incubating for 10 min at 65°C. The resulting 100 µl fraction (IP fraction) was incubated at 65°C overnight to reverse formaldehyde cross-links, in the presence of proteinase K (0.4 mg/ml). An aliquot of the cross-linked chromatin was treated as above, but in the absence of antibody (NA fraction), and the first supernatant was saved as the Input fraction. The DNA (from IP, NA and Input samples) was purified with PCR purification kit (Qiagen, Valencia, CA, USA) and used for PCR analysis of the target gene. Analysis of constitutively expressed *β-Actin* and of repressed *α-Actin* genes were used, respectively, as positive and negative controls of the experiment. The entire experiment was repeated at least three times.

To study nucleosome positioning as well as to detect histone modifications or modifier binding at the level of single nucleosomes, we employed the Nuc-ChIP technique. To do this, the sonication step of the standard ChIP protocol was replaced by extensive micrococcal nuclease digestion (see below) of isolated cross-linked nuclei. The resulting chromatin fragments are thus enriched in mononucleosomes, which were immunoprecipitated with the desired antibody. The detection of the immunoprecipitated DNA was carried out by PCR by using 1:500 dilutions of the Input and 1:30 dilutions of the IP and NA fractions. The integrated density of the electrophoretic bands (obtained by ImageJ software) of IP fraction was subtracted from that of the NA fraction, and the result normalized by that of the Input fraction. The entire experiments were repeated at least three times.

For the sequential ChIP analysis of histone epigenetic marks colocalization, aliquots from the mononucleosome-enriched chromatin fragments (equivalent to 100 µg DNA) were incubated with the first antibody and, after washings steps, the immunocomplexes were eluted by adding 75 µl TE buffer supplemented with 10 mM DTT and incubating for 30 min at 37°C. The resulting fractions were diluted 20 times with sequential ChIP dilution buffer (150 mM NaCl, 0.1% SDS, 1% Triton X-100, 1 mM EDTA, 20 mM Tris–HCl, pH 8.0), supplemented with protease inhibitor cocktail. Afterwards, the diluted samples were reimmunoprecipitated with a second antibody, and the standard procedure was followed. The sequential ChIP results were always confimed by changing the order in which the antibodies were used. To subtract the background signal to the IP samples, the integrated density of the electrophoretic bands of a parallel sample in which first antibody is replaced by IgG was used.

The antibodies used in our experiments were: α-CBP (sc-369), α-GCN5 (sc-303), α-PCAF (sc-8999), α-P300 (sc-584), α-mSIN3A (sc-994), α-NCoR (sc-8994), α-BRM (sc-710), α-BRG1 (sc-0768), α-MTA1 (sc-0813), α-SP1 (sc-59), α-CREB (sc-186), α-C/EBPβ (sc-150) and α-RNApol II (sc-899) from Santa Cruz; α-SNF2H (ab3749), α-H3K9ac (ab12181-50), α-H3K27ac (ab4729-100), α-H3K4me3 (ab1012-100), α-H4R3me2 (ab5823) and α-H3 (ab1791-22) from Abcam; α-H3K14ac (07-353), α-H4K5ac (07-327) and α-H3K9me2 (05-685) from Upstate-Millipore.

The primer pairs used for RNApol ChIP were: *Gas1,* forward 5′-CGAACAATGCAGGTCCACCAAG-3′, reverse 5′-TCGCACACGCAGTCGTTGAG-3′, amplicon position +937/+1209 and size 273 bp; *α-Actin,* forward 5′- GAGAAGATCTGGCACCACAC-3′, reverse 5′- CCCAGAATCCAACACGATC-3′, amplicon position +1325/+1759 and size 434 bp; *β-Actin,* forward 5′-TGTGCTGTCCCTGTATGCCTC-3′, reverse 5′-GGCCATCTCCTGCTCGAAG-3′, amplicon position +1993/+2263 and size 270 bp. The primer pairs used for standard ChIP were: *Gas1,* forward 5′- AACAACAGGCTGGACCAATAGC-3′, reverse 5′- ATGCATGCATAGAAAAGCAAACAACA-3′, amplicon position −252/−78 and size 174 bp; *α-Actin,* forward 5′-CACCTGACCACAGGGCTACC-3′, reverse 5′- AACTGGCTCCAAGGCTCACG-3′, amplicon position −484/−257 and size 227 bp; *β-Actin,* forward 5′-TCTGGCTTTCCGGCTATTGC -3′, reverse 5′- AGTTTTGGCGATGGGTGCTG-3′, amplicon position −540/−234 and size 306 bp.

### Nucleosome positioning by micrococcal nuclease protection assay (MNP)

To estimate the position of nucleosomes, isolated nuclei from cross-linked cells were incubated with micrococcal nuclease to obtain mononucleosomes. After purification of the mononucleosomal DNA, PCR analysis were performed using primers that amplified short tiled fragments covering the region comprised between (500 and +300 bp relative to the transcription start site (TSS) of Gas1. Consequently, amplification products were expected at regions in which a nucleosome was protecting DNA from nuclease digestion.

Nuclei were obtained from cross-linked livers, as described above, and nuclear pellets were resuspended in RSB buffer for micrococcal nuclease digestion. 7×108 nuclei were centrifuged at 2,000xg for 3 min at 4°C and washed with 1 ml of ice-cold RSB buffer [10 mM NaCl, 3 mM MgCl2, 1 mM CaCl2, 10 mM β-mercaptoethanol, 0.15 mM spermine, 0.5 mM spermidine, 2 µl/ml protease inhibitor cocktail (Sigma), 50 mM Tris-HCl pH 7,5]. After collecting nuclei by centrifugation, the pellets were resuspended in 0.5 ml RSB buffer without protease inhibitors, and incubated with 150 units of micrococcal nuclease (Roche Applied Science, Mannheim, Germany) for 30 min. Reaction was stopped by adding 1/10 volume EDTA 100 mM and putting the samples on ice. Nuclei were incubated at 65(C for 2 h to reverse cross-links in the presence of proteinase K (50 µg/ml). Finally, DNA was extracted and purified by phenol-chloroform extraction and precipitated with ethanol. The DNA pellet was resuspended in 100 µl of TE buffer and incubated 1 h at 37(C with 5 µg/ml RNAse A. The DNA from the mononucleosomal band was purified after running in 2% agarose gel by GenClean kit according to the manufacturer's instructions. Afterwards, DNA was used for PCR analysis, after quantifying by absorbance at 260 nm. The Input sample (an aliquot of the samples randomly fragmented by sonication to mononucleosomal size) was included to normalize the results for each primer pair in the PCR reaction. The entire experiments were repeated at least three times.

The primer pairs used for nucleosomal positioning were: amplicon (448, forward 5(-GGGCGGAGGAAGGGAAC-3(, reverse 5(-CACTCTTCAGGAGGCTGGGATAC-3(, size 120 bp; amplicon (329, forward 5(-TATCCCAGCCTCCTGAAGAGTG-3(, reverse 5(-CAGGCATTCAGGGCTTGAAA-3(, size 82 bp; amplicon (285, forward 5(- CCTGAATGTGAGCTGCCCA-3(, reverse 5(-GGTCCAGCCTGTTGTTGTTGA-3(, size 97 bp; amplicon (211, forward 5(-AACAACAGGCTGGACCAATAGC-3(, reverse 5(-TTCTCCTTTCTCCACTCTCCGG -3(, size 82 bp; amplicon (134, forward 5(- GGAGAGTGGAGAAAGGAGAAAG-3(, reverse 5(-ATGCATGCATAGAAAAGCAAAC AACA-3(, size 112 bp; amplicon (50, forward 5(- TTGTTTGTTTGCTTTTCTATGCATG-3(, reverse 5(-CCGGCTGCGGACTAGCT-3(, size 112 bp; amplicon 15, forward 5(-TCCCGGCCCACTTTTGTAT-3(, reverse 5(- GGAGGACCCCGAAACTCG-3(, size 121 bp; amplicon 125, forward 5(- GTTTCGGGGTCCTCCCTG-3(, reverse 5(- CGTAGCACTTCGCAGCTCTG-3(, size 127 bp; amplicon 187, forward 5(-AGGGGACCAAGCGTCCTG-3(, reverse 5(- AGGCGCCTGCAGACAG-3(, size 114 bp; amplicon 242, forward 5(-GCCAGAGCTGCGAAGTGCTAC-3(, reverse 5(-GAGGCGCTCAGTGCCGTTC-3(, size 151 bp.

For Nuc-ChIP and sequential-ChIP assays to detect histone modification marks, and binding of chromatin modifier and remodeller complexes to single nucleosomes, amplicon −211 (for nucleosome N-1) and amplicon +125 (for nucleosome N+1) were used.

## Supporting Information

Figure S1 *Gas1* promoter occupation by chromatin remodelling complexes after PH. The samples immunoprecipitated with indicated antibodies were analyzed by PCR using primers of the *Gas1* promoter region (Top panel). The integrated density of electrophoretic bands, obtained by ImageJ software analysis of the PCR signals, from the NA fraction was subtracted from that of IP fraction and normalized by that of input fraction. These images are representative of at least three different experiments.
(TIF)

Figure S2 *Gas1* promoter occupation by transcription factors after PH. The samples were processed as described in Figure S1.
(TIF)
