# Supplementary figures and images for: Epigenetic Transcriptional Regulation of the Growth Arrest-Specific gene 1 (Gas1) in Hepatic Cell Proliferation at Mononucleosomal Resolution
Source: PLoS One. 2011 Aug 9;6(8):e23318. doi: 10.1371/journal.pone.0023318 (PMC3153484; doi:10.1371/journal.pone.0023318)

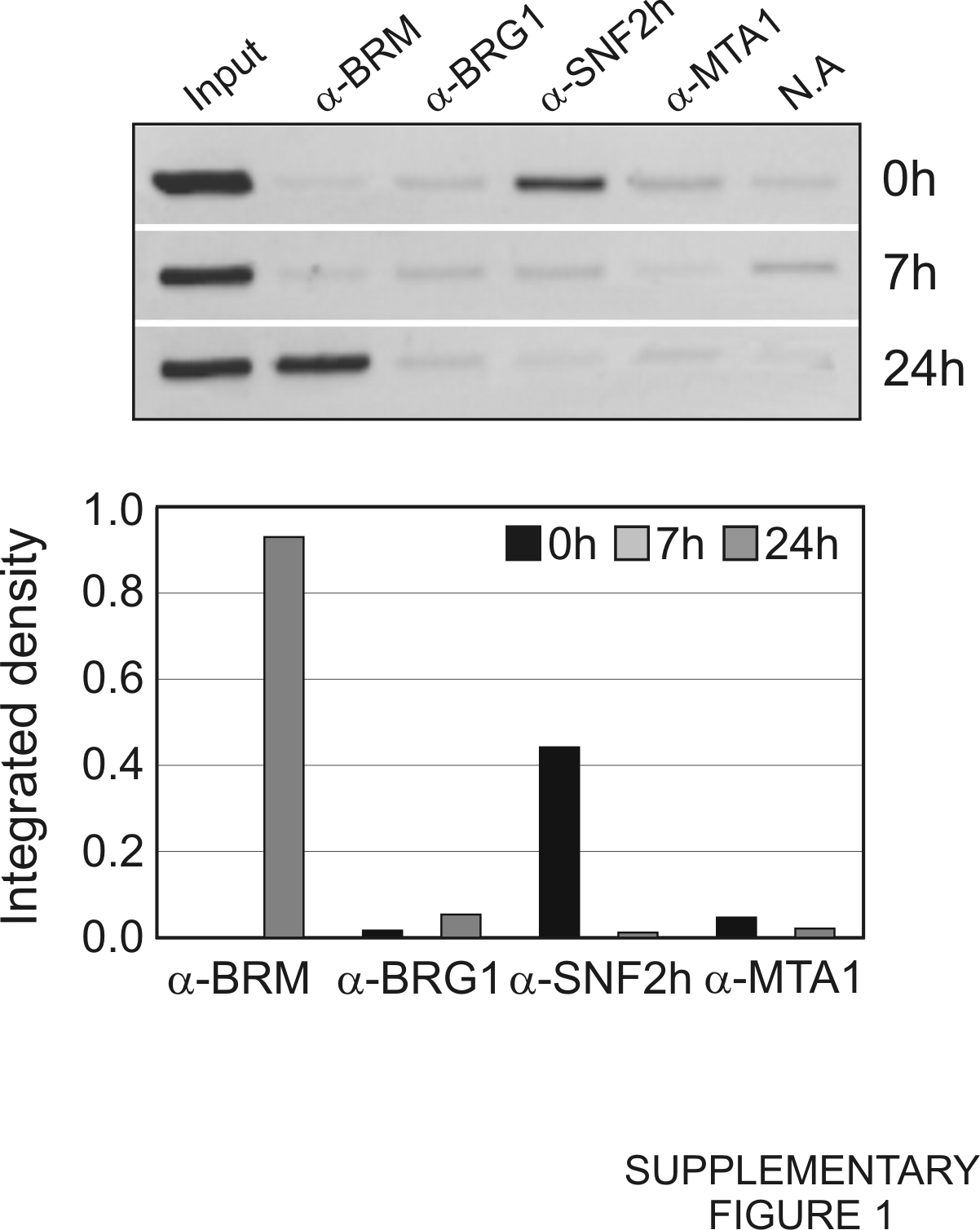

Supplement: Figure S1 — Gas1 promoter occupation by chromatin remodelling complexes after PH. The samples immunoprecipitated with indicated antibodies were analyzed by PCR using primers of the Gas1 promoter region (Top panel). The integrated density of electrophoretic bands, obtained by ImageJ software analysis of the PCR signals, from the NA fraction was subtracted from that of IP fraction and normalized by that of input fraction. These images are representative of at least three different experiments. (TIF) [file pone.0023318.s001.tif]

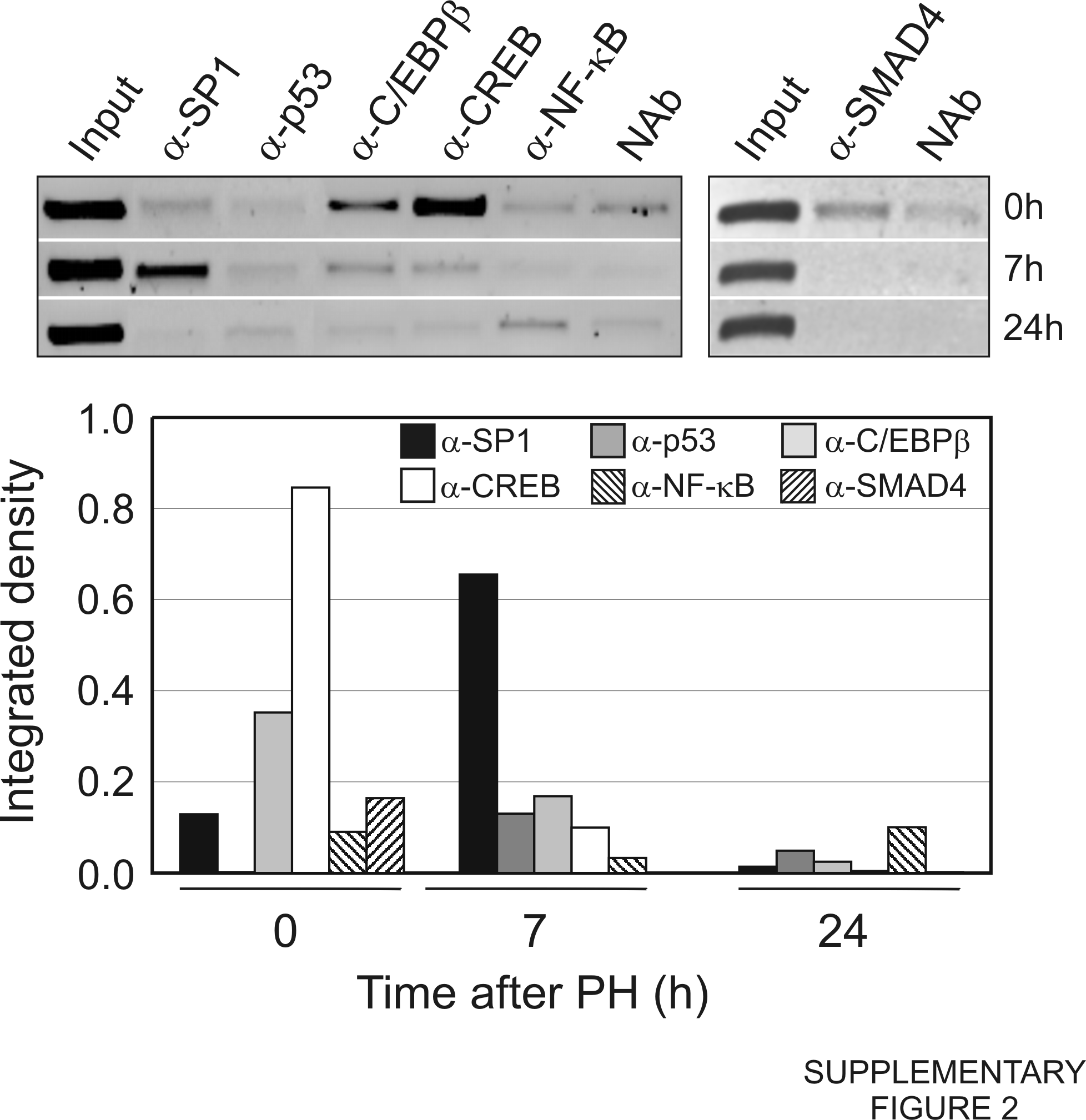

Supplement: Figure S2 — Gas1 promoter occupation by transcription factors after PH. The samples were processed as described in Figure S1. (TIF) [file pone.0023318.s002.tif]
